# Detection of *Helicobacter pylori* in Various Types of Vegetables and Salads

**DOI:** 10.5812/jjm.10013

**Published:** 2014-05-01

**Authors:** Shahrzad Atapoor, Farhad Safarpoor Dehkordi, Ebrahim Rahimi

**Affiliations:** 1 Faculty of Agriculture and Natural Sciences, Shahrekord Branch, Islamic Azad University, Shahrekord, IR Iran; 2 Young Researchers and Elites Club, Shahrekord Branch, Islamic Azad University, Shahrekord, IR Iran; 3 Department of Food Hygiene and Public Health, College of Veterinary Medicine, Shahrekord Branch, Islamic Azad University, Shahrekord, IR Iran

**Keywords:** *Helicobacter pylori*, Vegetables, Culture, Polymerase Chain Reaction

## Abstract

**Background:**

There is a possibility for the presence of *Helicobacter pylori* in vegetables due to their close contact with polluted water, soil and feces.

**Objectives:**

This study was carried out to detect the presence of *H. pylori* in vegetables and salads in Iran.

**Materials and Methods:**

In total, 460 vegetable and salad samples were collected and transferred immediately to the laboratory. All samples were cultured and tested for the presence of *H. pylori* using the Polymerase Chain Reaction technique.

**Results:**

The results showed that 44 of 460 samples (9.56%) were positive for *H. pylori* using the culture method. The Polymerase Chain Reaction technique showed that 50 of 460 samples (10.86%) were positive for *H. pylori*. Un-washed leek, traditional salad, un-washed basil and un-washed lettuce were the most commonly contaminated samples. The presence of the bacteria in various vegetables was statistically significant (P < 0.05).

**Conclusions:**

Vegetables are a new source of *H. pylori* and accurate washing of vegetables improves such contaminations.

## 1. Background

Vegetables and salads are rich and comparatively cheaper source of vitamins. Consumption of these food sources provides taste, palatability, increases appetite and provides fiber for digestion and prevents constipation. Vegetables are in contact with soil, polluted water, animal manure and even stool. Therefore, they can easily become contaminated. A previous study showed that soil, water, animal manure and stool (1) are the main sources of *Helicobacter pylori*. *H. pylori* is a microaerophilic Gram negative bacteria with a curved spiral shape which is known as a causative agent of type B gastritis, peptic ulcer disease, gastric adenocarcinoma and mucosa associated lymphoid tissue lymphoma (2). The bacteria has been classified as a Class I carcinogen by the World Health Organization (3). The prevalence of infection is typically higher in developing countries (> 80%) and lower in developed nations (< 40%) with a declining pattern worldwide (4, 5).

Prescription of antibiotics is the main protocol for treatment of diseases caused by *H. pylori *(6). However, antibiotic therapy fails in about 20% of the patients (5), mainly due to antibiotic resistance (7). During the last two decades, the role of *H. pylori* as a potential pathogen in both human and veterinary medicine has been investigated intensively and evidence suggests possible zoonotic transmission of animal helicobacters to humans.

## 2. Objectives

The epidemiology and prevalence of *H. pylori* in food sources, especially vegetables and salads, is essentially unknown. Therefore, the present study was carried out in order to detect *H. pylori* in various types of traditional, commercial, washed and un-washed vegetables and salads in Iran.

## 3. Materials and Methods

### 3.1. Samples and Isolation of *H. pylori*

A total of 460 vegetable and salad samples were collected from supermarkets and groceries of various parts of Iran (Table 1). The samples were processed within an hour of collection. Samples were homogenized and 25 mL of each sample was added to 225 mL of Columbia blood agar (Oxoid, UK) supplemented with 5% horse serum (Sigma, St. Louis, MO, USA) and colistinme than esulfonate (30 mg/L), cycloheximide (100 mg/L), nalidixic acid (30 mg/L), trimethoprim (30 mg/L), and vancomycin (10 mg/L) (Sigma, St. Louis, MO, USA) and incubated for seven days at 37°C with constant shaking under microaerophilic conditions. Next, 0.1 mL of the enrichment selective broth was plated onto Columbia blood agar (Oxoid, UK) supplemented with 5% of defibrinated horse blood and 30 mg/L colistinmethanesulfonate, 100 mg/L cycloheximide, 30 mg/L nalidixic acid, 30 mg/L trimethoprim, and 10 mg/L vancomycin (Sigma, St. Louis, MO, USA) (8) and incubated for seven days at 37°C under microaerophilic conditions. Suspected colonies were identified as *H. pylori* based on the method described by Dunn et al. (9). For comparison, a reference strain of *H. pylori* (ATCC 43504) was employed. The isolates were confirmed using the PCR assay.

**Table 1.** Distribution of *H. pylori* in Commercial and Traditional Salads and Washed and Un-washed Vegetables Using Culture and PCR Techniques ^a^

Type of Samples	Number of Samples	Positive Culture	Positive PCR	
**Salad**			
Commercial	30	1 (3.33)	1 (3.33)
Traditional	30	7 (23.33)	9 (30)
**Basil**			
Washed	20	-	-
Un-washed	20	4 (20)	5 (25)
**Radish**			
Washed	20	-	-
Un-washed	20	3 (15)	3 (15)
**Leek**			
Washed	20	1 (5)	1 (5)
Un-washed	20	6 (30)	7 (35)
**Spinach**			
Washed	40	1 (2.5)	1 (2.5)
Un-washed	40	4 (10)	4 (10)
**Lettuce**			
Washed	40	3 (7.5)	3 (7.5)
Un-washed	40	7 (17.5)	8 (20)
**Parsley**			
Commercial	40	1 (2.5)	1 (2.5)
Washed	40	2 (5)	2 (5)
Un-washed	40	4 (10)	5 (12.5)
**Total**	460	44 (9.56)	50 (10.86)

^a^ Data are presented in NO. (%).

### 3.2. DNA Extraction and PCR Amplification

DNA from 1 mL of each vegetable and salad sample was extracted by a DNA isolation kit for cells and tissues (Roche Applied Science, Germany, 11814770001), according to the manufacturer’s instructions. Extracted genomic DNA was amplified for the ureC gene and detected with specific primers HP-F: 5'-GAATAAGCTTTTAGGGGTGTTAGGGG-3’, HP-R: 5'GCTTACTTTCTAACACTAACGCGC-3'. The gene product was 294 bp. The PCR conditions and temperatures were based on the Rahimi and Kheirabadi protocol (10). Samples inoculated with *H. pylori* were used as positive controls.

### 3.3. Statistical Analysis

Data were transferred to Microsoft Excel spreadsheet (Microsoft Corp., Redmond, WA, USA) for analysis. Using SPSS 16.0 statistical software (SPSS Inc., Chicago, IL, USA), Chi-square test and Fisher’s exact two-tailed test analysis were performed and differences were considered significant with P < 0.05.

## 4. Results

**Figure 1.**
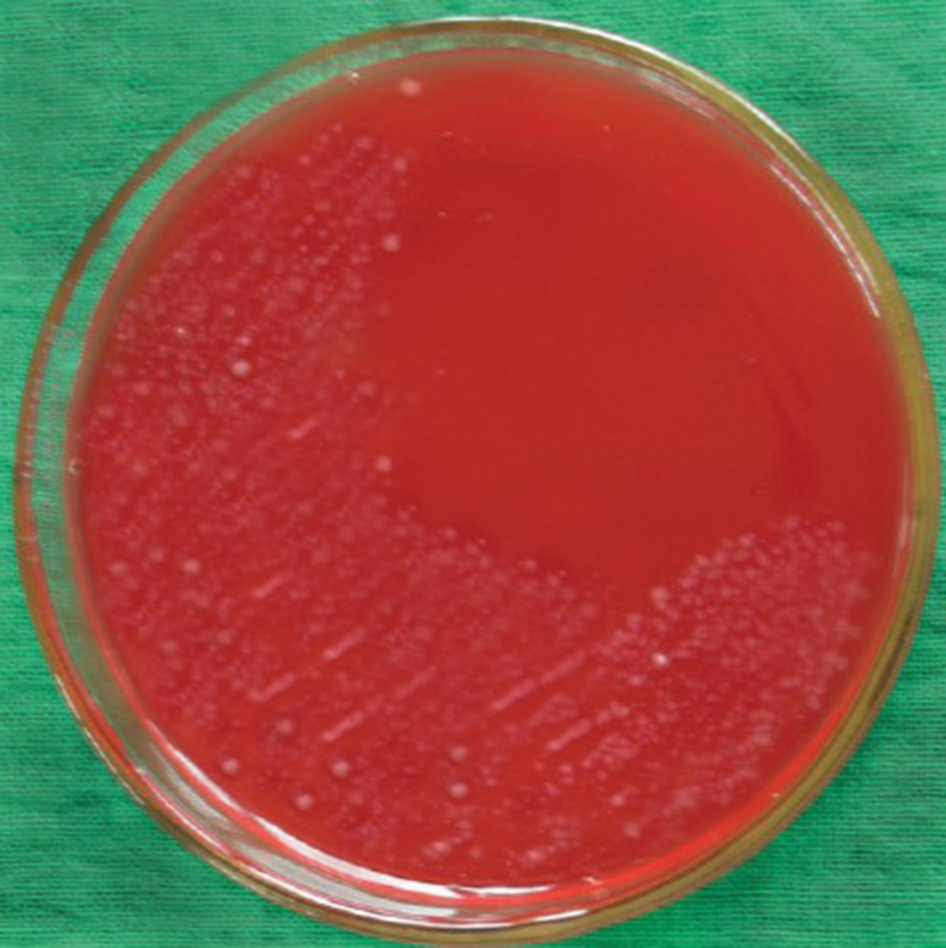
The colonies of *H. pylori* on Columbia Blood Agar Medium

Table 1 presents the distribution of *H. pylori* isolated from various types of vegetable and salad samples in Iran. In this study, 44 of 460 samples (9.56%) were found to be contaminated with *H. pylori* using the culture method (Figure 1). There were significant differences (P < 0.05) between the prevalence rates of *H. pylori* in commercial and traditional salads as well as between washed and un-washed vegetables. The PCR technique showed that 50 of 460 samples (10.86%) were contaminated with *H. pylori* (Table 1). No significant differences were observed between the abilities of culture and PCR techniques for detection of *H. pylori* in vegetable and salad samples. The most commonly contaminated vegetables were un-washed leek (35%), followed by un-washed basil (25%) and un-washed lettuce (20%).

## 5. Discussion

Several studies have addressed the role of food in the transmission of *H. pylori *(10, 11). Moreover, nowadays there is an increasing demand for minimally processed vegetables packed under a modified atmosphere (12). Several studies have confirmed the high presence of *H. pylori* in pasteurized and sterilized food products (13, 14). Therefore, emphasis on hygiene can be an exceptional way for reducing the load of *H. pylori* in foods. Food products that have been analyzed thus far mainly include milk, meat and vegetables. Among these, milk products are the most studied while vegetables are rare (14).

Rahimi and Kheirabadi (10) reported that the *H. pylori *ure C gene was detected in 56 of 448 (12.5%) Iranian milk samples, including 19 cows (14.1%), 11 sheep (12.2%), nine goats (8.7%), two camels (3.6%) and 15 buffalo (23.4%) milk samples. However, it has been described that individuals who consume vegetables are more likely to acquire *H. pylori* (15). Also, the isolation of *H. pylori* is not always associated with raw milk. For instance a study on 440 raw sheep milk samples did not yield any *H. pylori* isolates (16). Besides, *H. pylori* can survive for short periods in milk (17). 

The association of the infection with consumption of raw vegetables is an additional indirect evidence for the presence of *H. pylori* in water used for irrigation of these vegetables (17, 18). A previous study indicated that poor quality water could represent an important vehicle for *H. pylori *transmission (19). In addition to water used for irrigation of vegetables, animal manure used for reinforcement of soil is an additional indirect evidence for the presence of *H. pylori* in vegetables. This bacteria has been isolated previously from cow’s fecal samples (20). Feces of animal and especially cows have been used for reinforced agricultural soil. Fujimura et al. (13) showed that the prevalence of *H. pylori* was 50% in cow feces and 38% in soil samples. Also, this bacteria has been isolated from various animal sources (21). Contact with cow feces is one of the main sources of vegetable contamination.

Another previous study showed that *H. pylori* survived for 72 hours in sanitized and up to 96 hours in sterilized vegetables (22, 23). Foods with water activity higher than 0.97 and pH ranging from 4.9 to 6.0 theoretically provide conditions for the survival of *H. pylori*. Also, the general lack of efficient sanitation in removing or killing pathogens on raw fruits and vegetables may contribute to harbor pathogens (12). *H. pylori* is unlikely to grow on most food products, but it is able to survive in a low acid and high moisture environment for extended periods of time, especially if refrigerated. As far as we know, vegetables grow in high moisture soil, which can allow *H. pylori *development for a long duration of time.

This is the first paper to report on the contamination of basil, spinach, salad, parsley, leek and radish by *H. pylori* in Iran. Proof of the ability of *H. pylori* to survive in common foods supports the hypothesis that primary contamination of a food product (animal reservoir) or secondary contamination due to inappropriate handling (human reservoir) can be a vehicle for *H. pylori* transmission. Polluted water, feces, animal manure and even soil are the main resources for contamination of vegetables with *H. pylori*. The results of this study showed that *H. pylori* have a higher presence in un-washed vegetables and traditional salads. Therefore, pasteurization, sterilization and accurate washing can reduce the microbial load of vegetables.
